# Supplementary material for: Causal interplay between lactose intolerance and gut microbiota: a combined bidirectional Mendelian randomization and in vivo validation study
Source: Front Nutr. 2026 Jun 1;13:1803337. doi: 10.3389/fnut.2026.1803337 (PMC13265576; doi:10.3389/fnut.2026.1803337)
Supplement: Supplementary file 1 [file Data_Sheet_1.zip › supplementary materials/Forward/leave one out cross validation/ebi-a-GCST90027775.finngen_R12_E4_LACTONAS.pdf]

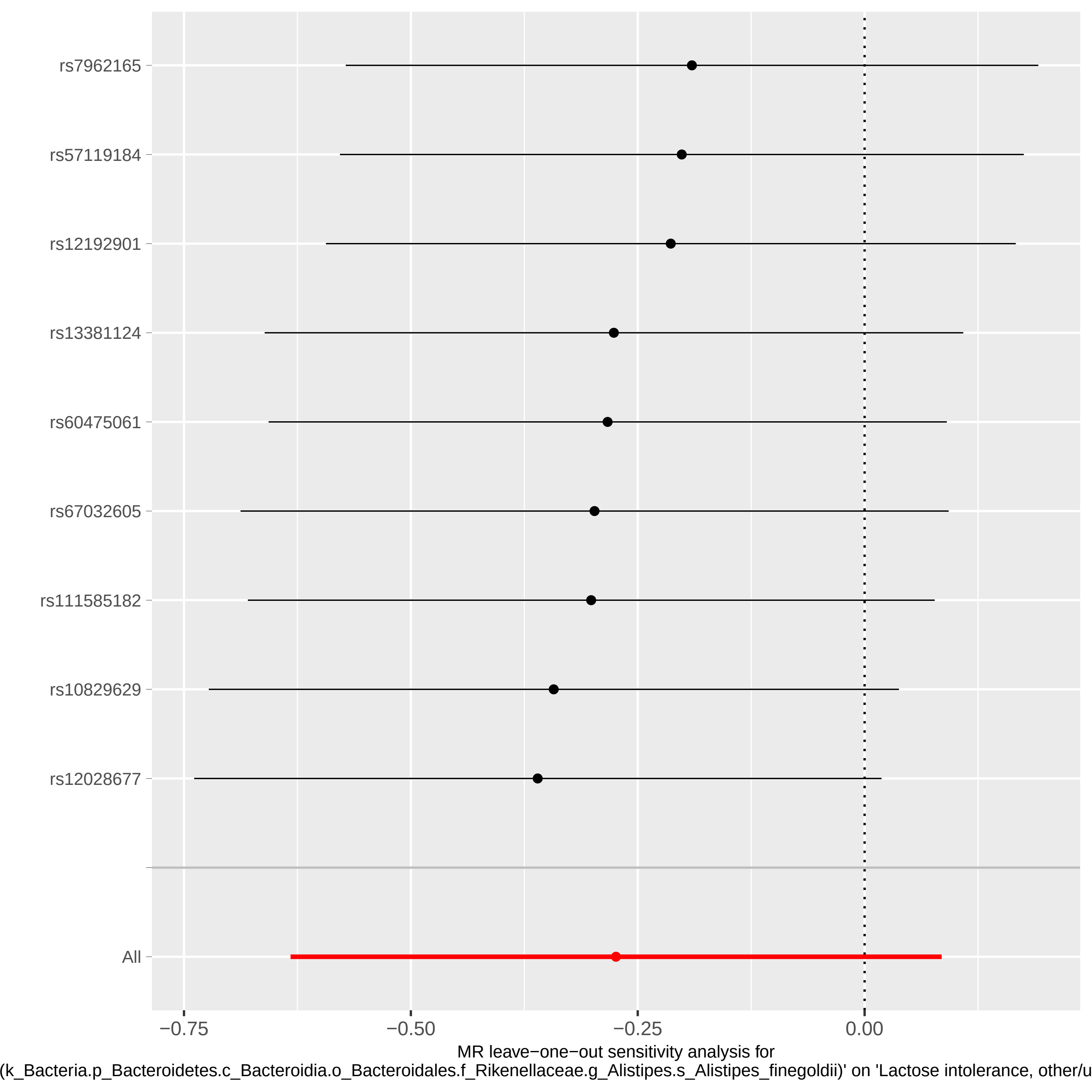
rs7962165
rs57119184
rs12192901
rs13381124
rs60475061
rs67032605
rs111585182
rs10829629
rs12028677
All
−0.75
−0.50
−0.25
0.00
MR leave−one−out sensitivity analysis for
(k_Bacteria.p_Bacteroidetes.c_Bacteroidia.o_Bacteroidales.f_Rikenellaceae.g_Alistipes.s_Alistipes_finegoldii)' on 'Lactose intolerance, other/u
